# Metabolic Dysfunction-Associated Fatty Liver Disease in Taiwanese Patients with Inflammatory Bowel Disease: A Study in Patients with Clinical Remission

**DOI:** 10.3390/diagnostics13203268

**Published:** 2023-10-20

**Authors:** Shun-Wen Hsiao, Ting-Chun Chen, Pei-Yuan Su, Chen-Ta Yang, Siou-Ping Huang, Yang-Yuan Chen, Hsu-Heng Yen

**Affiliations:** 1 Division of Gastroenterology, Changhua Christian Hospital, Changhua 500, Taiwan; 2 Division of Endocrinology and Metabolism, Cheng Ching Hospital, Taichung 400, Taiwan; 3 Department of Post-Baccalaureate Medicine, College of Medicine, National Chung Hsing University, Taichung 402, Taiwan; 4 Division of Gastroenterology, Yuanlin Christian Hospital, Changhua 500, Taiwan; 5 Department of Hospitality Management, MingDao University, Changhua 523, Taiwan; 6 Artificial Intelligence Development Center, Changhua Christian Hospital, Changhua 500, Taiwan

**Keywords:** inflammatory bowel disease, fatty liver, Crohn’s disease, ulcerative colitis

## Abstract

The prevalence of inflammatory bowel disease (IBD) has increased worldwide. The prevalence of metabolic dysfunction associated fatty liver disease (MAFLD) has also risen. However, there is limited research on the connection between MAFLD and IBD in the Asian population. This study aims to analyze the prevalence and clinical significance of MAFLD in Taiwanese IBD patients with clinical remission. We retrospectively analyzed IBD patients who received transient elastography for liver fibrosis and controlled attenuation parameter evaluation for liver steatosis. This study enrolled 120 patients with IBD, including 45 Crohn’s disease (CD) and 75 ulcerative colitis (UC). MAFLD prevalence in IBD was 29.2%. Patients with MAFLD had a shorter disease duration (2.8 years vs. 5.3 years, *p* = 0.017), higher alanine aminotransferase levels (24 U/L vs. 17 U/L, *p* = 0.003), a lower estimated glomerular filtration rate (91.37 mL/min/1.73 m^2^ vs. 103.92 mL/min/1.73 m^2^, *p* = 0.004), and higher γ-glutamyl transferase (γ-GT) (24 mg/dL vs. 13 mg/dL, *p* < 0.001). The prevalence of significant fibrosis in IBD with MAFLD was 17.1%. Significant fibrosis was found in older age (58.5 years vs. 40 years, *p* = 0.004) and the high type 2 diabetes mellitus proportion (50.0% vs. 10.3%, *p* = 0.049). A trend of longer disease duration was found in significant fibrosis (4.9 years vs. 1.6 years, *p* = 0.051). The prevalence of MALFD in IBD was 29.2%. and 17.1% of them had significant fibrosis. In addition to the intestinal manifestation, the study findings remind clinicians that they should be aware of the possibility of hepatic complications for IBD patients.

## 1. Introduction

Inflammatory bowel diseases (IBDs), including Crohn’s disease (CD) and ulcerative colitis (UC), are chronic inflammatory disorders mostly affecting the gastrointestinal tract causing immunologic dysregulation with genetic factors, gut microbiota, and environmental factors [1]. Recently, IBD prevalence has increased globally, initially in Western countries as well as in Asian countries, including Taiwan, which has likely been related to environmental factors and the Westernized diet and lifestyle [2,3].

A similar increased prevalence was noted in nonalcoholic fatty liver diseases (NAFLD) [4,5]. NAFLD, also known as metabolic dysfunction-associated fatty liver disease (MAFLD), was defined as intracellular fat deposition in the liver of >5% without excessive alcohol consumption, toxin, or a viral cause of hepatitis, including a hepatitis B virus and hepatitis C virus infection [6]. MAFLD, which was first introduced in 2019 with an expert’s consensus that precisely represented the pathogenesis of fatty liver, was associated with metabolic dysfunction, including overweight, obesity, type 2 diabetes mellitus (T2DM), or normal weight with metabolic syndrome or lipid disorders [7]. MAFLD was also associated with IBD, with marked prevalence in different regions. A meta-analysis reported the prevalence of NAFLD in IBD at 36.9%, 17.2%, and 11.8% in European, Western Pacific, and Eastern Mediterranean countries, respectively [8]. Meanwhile, MAFLD-associated liver fibrosis in patients with IBD, which was represented in few studies, demonstrated an estimated prevalence of 1.2%–16% which differed due to different methods, such as noninvasive biomarkers, a Fibrosis-4 index, transient elastography (TE), or a histopathology diagnosis [9,10,11].

The risk factors of developing MAFLD in patients with IBD remain undetermined [10]. Some studies have supported traditional risk factors, such as T2DM, weight gain, or obesity, to contribute to MAFLD development in patients with IBD [6,12,13]. Other studies have highlighted the involvement of disease activity, duration, and drug-induced liver injury in MAFLD progression [9,14].

This study aims to analyze the prevalence of MAFLD and fibrosis in patients with IBD in our cohort by TE and to identify the associated risk factors with liver steatosis and fibrosis.

## 2. Materials and Methods

### 2.1. Inclusion Criteria

**Figure 1:**
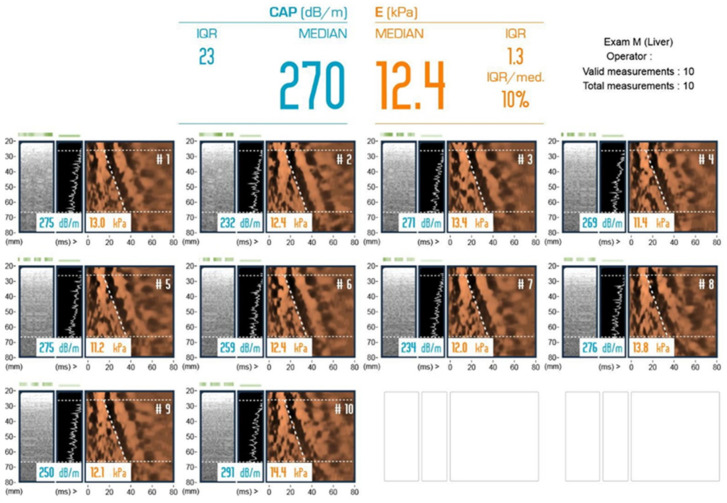
The LSMs and CAP measurements were performed using FibroScan (Echosens, Paris, France) by an experienced operator who has performed more than 5000 FibroScan examinations. The finding LSM:12.4 kPa/CAP:270 dB/m was judged as S2 and F3.

We retrospectively analyzed patients with IBD from January 2019 to April 2023 at our institution. We involved patients with IBD from January 2019 in an in-hospital liver disease surveillance program, including abdominal ultrasound examination and TE encompassing liver stiffness measurements (LSM) for evaluating liver fibrosis and controlled attenuation parameters (CAPs) for liver steatosis (Figure 1). The liver stiffness was divided into four stages with cut-off values of LSM for staging as F0–1, F2, F3, and F4 at <7.2 kPa, <9.7 kPa, <12.5 kPa, and ≥12.5 kPa, respectively, based on previous literature [15,16]. F2, F3, and F4 were defined as significant fibrosis, advanced fibrosis, and standard for cirrhosis, respectively. We clarify steatosis in the liver as the median value of the CAP of ≥248 dB/m and staging as follows: CAP of ≥248 dB/m, ≥268 dB/m, and ≥288 dB/m as S1, S2, and S3, respectively [15].

Regular abdominal ultrasound for steatosis was defined as normal, mild, moderate, or severe fatty liver and judged by a hepatologist [17]. Body mass index (BMI) was defined by the World Health Organization consensus for the Asia group as underweight (<18.5 kg/m^2^), normal weight (18.5–23 kg/m^2^), overweight (23–27.5 kg/m^2^), and obesity (≥27.5 kg/m^2^) [18]. Inclusion criteria were definite CD or UC diagnosis and National Health Insurance-certified major illness at age >20 years old and having received a transabdominal ultrasound and TE examination with LSM and CAP at clinical remission status. Lack or failure of LSM and CAP examination were excluded. We retrospectively review the chart for variable factors, such as age, gender, BMI, bowel resection history, laboratory measurements, such as white blood cell (WBC) counts, hemoglobin (Hb), aspartate aminotransferase (AST), alanine aminotransferase (ALT), γ-glutamyl transferase (γ-GT), alkaline phosphatase (ALK-P), fasting glucose, fasting insulin, glycated hemoglobin (HbA1c), creatine, triglyceride (TG), low-density lipoprotein cholesterol (LDL-C), total cholesterol, high-density lipoprotein cholesterol (HDL-C), albumin, C-reactive protein (CRP), erythrocyte sedimentation rate (ESR), and medical history of hypertension, T2DM, and biologic products used.

### 2.2. Metabolic Dysfunction-Associated Fatty Liver Diseases

We clarify steatosis in the liver as the median value of a CAP at ≥248 dB/m [15]. The MAFLD diagnosis was defined as hepatic steatosis and concomitant with T2DM, BMI of ≥23 kg/m^2^, or presenting with at least two of the following metabolic abnormalities: waist circumference of ≥90 in the male and ≥80 in the female, blood pressure of ≥130/85 mmHg, TG of ≥150 mg/dLl, HDL-C of <40 mg/dL in the male and <50 mg/dL in the female, or under controlled medication for blood pressure and dyslipidemia, prediabetes, fasting glucose of >100 mg/dL or HbA1c of 5.7%–6.4%, homeostasis model assessment of insulin resistance score (HOMA-IR) of ≥2.5 in the female and serum CRP level of >2 mg/L.

### 2.3. Statistical Analysis

The demographic and other clinical data of patients were expressed as frequency (%), median (interquartile range [IQR], 25th–75th percentile), or mean ± standard deviation (SD). The one-sample Kolmogorov–Smirnov Test evaluated the distribution of continuous variables. We used a Mann–Whitney U test or the Student’s t-test for continuous data. We used Fisher’s exact test or chi-square test for categorical data. We used multivariable logistic regression analysis to analyze factors associated with the risk of MAFLD. We discarded diagnostic criteria for MAFLD and selected variables with *p*-values of <0.05 from the crude model with backward elimination to enter multivariate adjustment. The statistical significance was defined as *p*-values of <0.05 with two sides. We used an IBM Statistical Package for the Social Sciences version 22.0 (IBM Corp., Armonk, NY, USA) for statistical analyses.

## 3. Results

### 3.1. Baseline Characteristics of IBD, including CD and UC

This study enrolled 120 patients with IBD, including 45 with CD and 75 with UC, as shown in Table 1. The male was more predominant in IBD accounting for 67.5%, including 73.3% with CD and 64% with UC. The median age at diagnosis was 43.5 years, which was younger for CD than for UC (37 years vs. 45 years, *p* = 0.089). Patients with CD demonstrated more gallstone (26.7% vs. 6.7%, *p* = 0.002) and bowel resection history (46.7% vs. 2.7%, *p* < 0.001). BMI, abdominal circumference, AST, ALT, fasting glucose, and HbA1c demonstrated no difference. However, CD had a significantly higher neutrophil-to-lymphocyte ratio (3.42 vs. 2.53, *p* = 0.027), low albumin (4.2 g/dL vs. 4.4 g/dL, *p* = 0.023), a higher CRP level (0.24 mg/dL vs. 0.12 mg/dL, *p* = 0.045), and higher ALK-P (72 vs. 56 U/L, *p* = 0.003). Furthermore, liver steatosis, the CAP, and fibrosis, including Fib-4 or LSM, demonstrated no difference.

### 3.2. Comparison of MAFLD with Non-MAFLD

Liver steatosis was detected in 39 patients as shown in Table 2. The definition of MAFLD was fulfilled in 35 patients, and the prevalence was 29.2% in patients with IBD. MAFLD patients had a higher BMI (26.3 kg/m^2^ vs. 21.5 kg/m^2^, *p* < 0.001), greater abdominal circumference (93 cm vs. 76 cm, *p* < 0.001), higher TG (132 mg/dL vs. 74 mg/dL, *p* < 0.001), lower HDL (44 mg/dL vs. 49 mg/dL, *p* = 0.026), higher HOMA-IR (1.91 vs. 0.9, *p* = 0.003), and higher LDL (117 mg/dL vs. 98 mg/dL, *p* = 0.016) compared with non-MAFLD patients due to enrolled factors for diagnosis. However, patients with MAFLD had a shorter disease duration (2.8 years vs. 5.3 years, *p* = 0.017), higher ALT levels (24 U/L vs. 17 U/L, *p* = 0.003), a lower estimate of the glomerular filtration rate (eGFR) (91.37 mL/min/1.73 m^2^ vs. 103.92 mL/min/1.73 m^2^, *p* = 0.004), and higher γ-GT (24 mg/dL vs. 13 mg/dL, *p* < 0.001). Furthermore, MAFLD patients demonstrated a higher LSM value (5.3 kpa vs. 4.9 kpa, *p* = 0.024) and significant fibrosis (17.1% vs. 2.4%, *p* = 0.008). Otherwise, WBC counts (6.9 × 10^3^/μL vs. 5.7 × 10^3^/μL, *p* = 0.090), CRP (0.17 mg/dL vs. 0.11 mg/dL, *p* = 0.161), or ESR (11 mm/h vs. 12 mm/h, *p* = 0.943) demonstrated no difference.

### 3.3. Multivariate Analysis of Factors Associated with MAFLD

Table 3 shows the multivariate analysis of factors associated with MAFLD. We excluded metabolic factors, such as BMI, lipid profile, CRP, fasting glucose, or HOMA-IR, because they are conditions for MAFLD diagnosis. We selected crude model variables with *p*-values of <0.05 into a multivariate adjustment. MAFLD was associated with higher Hb (adjusted odds ratio [aOR]: 1.91, 95% confidence interval [CI]: 1.17–3.13, *p* = 0.010), elevated γ-GT (aOR: 1.11, 95% CI: 1.02–1.21, *p* = 0.013), and significant liver fibrosis, namely F2, F3, and F4 (aOR: 31.25, 95% CI: 1.2–815.55, *p* = 0.039).

### 3.4. Factors Associated with Significant Fibrosis

**Figure 2:**
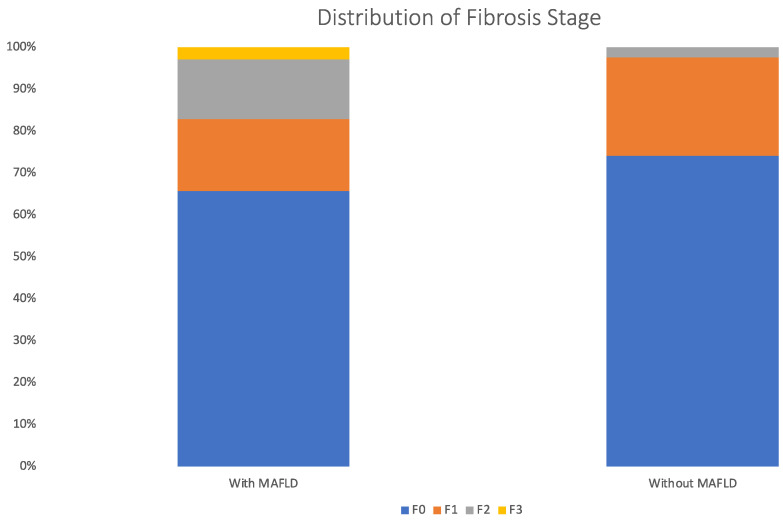
Distribution of liver fibrosis stage by LSM in patients with and without MAFLD.

**Figure 3:**
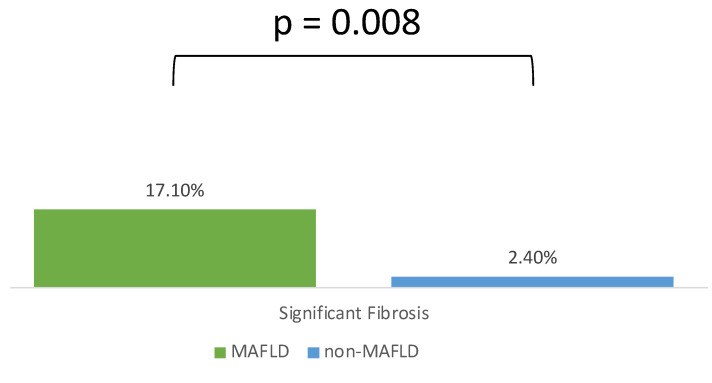
MAFLD patients have a higher proportion of significant fibrosis than those without MAFLD.

The distribution of the liver fibrosis stage estimated by LSM is illustrated in Figure 2. The prevalence of significant fibrosis (fibrosis stage ≥ F3) in patients with MAFLD was 17.1% compared with 2.1% in patients without MAFLD (*p* = 0.008) (Figure 3).

Table 4 shows the comparison of features of significant fibrosis among the MAFLD population. Significant fibrosis was associated with older age (58.5 years vs. 40 years, *p* = 0.004), a high T2DM history proportion (50.0% vs. 10.3%, *p* = 0.049), and lower platelet counts (195 × 10^3^/μL vs. 261 × 10^3^/μL, *p* = 0.049). Furthermore, we noticed the trend of a longer disease duration with significant fibrosis (4.9 years vs. 1.6 years, *p* = 0.051).

## 4. Discussion

The prevalence of MAFLD in patients with IBD in our cohort was 29.2%. Conditions for diagnosis, such as a higher BMI, insulin resistance, and metabolic syndrome, patients with MAFLD having higher ALT and γ-GT, lower eGFR and significant fibrosis were excluded from the study. MAFLD was associated with elevated γ-GT and significant fibrosis after being adjusted with multivariate analysis.

The prevalence of MAFLD in IBD was mostly analyzed in America and Europe and was 28.2% (95% CI: 22.2–34.3), and 36.9% (95% CI: 31.2–42.6), respectively [8]. A few studies addressed the prevalence of MAFLD in IBD in the Asia-Pacific region (Table 5). The MAFLD prevalence was 10.7% in patients with IBD in China, 16.7% in patients with IBD in South Korea, and 21.8% in CD patients in Japan [19,20,21]. The discordance of prevalence was affected by the measurement methods and regional differences. We had previously published and reported a MAFLD prevalence of 29.6% in IBD [10]. On this occasion, we introduced a new definition of MAFLD with the extended database which showed the prevalence of MAFLD in IBD at 29.2%. Liver steatosis was detected in 39 patients, and 90% of patients had metabolic disorders and were diagnosed with MAFLD. Our TE database does not contain any information regarding the prevalence of MAFLD in the general population for comparison, but our previous ultrasound-based report found that the prevalence of it in the Taiwanese population was 26% [22].

The patients gained weight and the prevalence of obesity was approximately 32.7% with more effective therapy, such as biologics and well-controlled disease with persistent remission [23]. Subsequently, the increased BMI in IBD was associated with more metabolic syndrome, NAFLD development, and liver fibrosis [24]. One recent meta-analysis revealed that increased age and higher BMI were associated with the risk of NAFLD development with aOR of 1.03 (95% CI: 1.01–1.05) and 1.27 (95% CI: 1.22–1.32), respectively [8]. However, a statistically significant increased risk of NAFLD development, including diabetes, hypertension, dyslipidemia, and surgical history, was not achieved, with aORs of 1.84 (95% CI: 0.86–2.83), 1.15 (95% CI: 0.25–2.06), 2.00 (95% CI: 0.00–4.48), and 1.22 (95% CI: 0.51–1.93), respectively. The statistically insignificant and wide 95% CI with a vertical line at 1.0 was due to the small number of studies. Some authors indicated metabolic syndrome as a risk factor for developing NAFLD in IBD as well as the general population [25]. However, one recent study involving two medical centers analyzed the risk factors of developing MAFLD in patients with IBD compared with healthy control matched by age, sex, T2DM, and BMI, which revealed IBD as a predictor for MAFLD development and liver fibrosis with an OR of 1.99 (*p* < 0.001) and 5.55 (*p* < 0.001), respectively [26]. Our cohort was lacking a controlled group for comparison. We performed multivariate adjustment after excluding diagnosed conditions for MAFLD, and MAFLD was associated with elevated γ-GT and significant fibrosis.

A few studies addressed liver fibrosis in patients with IBD with fatty liver. NAFLD prevalence with liver fibrosis differed. Veltkamp reported a prevalence of 8% in patients with IBD defined as TE of >7 kPa, and mostly in patients with CD [27]. Ritaccio reported a prevalence of 4% in IBD defined as an NAFLD fibrosis score (NFS) of >0.675 [28]. Bessissow reported a prevalence of 2.2% in IBD defined as FIB-4 of ≥2.67 [29]. A recent study in Korea reports a prevalence of 5.3% in IBD with FIB-4 of ≥1.45 [21]. Ritaccio reported that 16% of patients had progressed to a NFS during 5-year follow-ups. Despite a small cohort, 56 of 138, at 5-year follow-up, the author emphasized the result representing pattern of disease fluctuation [28]. Our cohort demonstrates a 17.1% prevalence of significant fibrosis, with TE of ≥7.2 kPa. This significant fibrosis was observed to be more common in older individuals and those with a higher portion of T2DM. Additionally, we observed a trend indicating that longer disease duration was associated with a higher likelihood of significant fibrosis. These findings may indicate that advancing age and prolonged disease duration may contribute to significant fibrosis development and progression. It also emphasizes the need for regular monitoring and follow-up over an extended period.

Our study had several limitations. First, this single-center study had a limited sample size which may affect the statistical power and precision of the results. Second, histopathology diagnosis, which remains a gold standard for the diagnosis of NAFLD and NASH, was lacking. Instead, we utilized TE as a more objective method to detect steatosis and fibrosis and employed a new inclusive criterion for MAFLD diagnosis to account for the possible impact of a concomitant with chronic hepatitis B and C infection. Third, we did not analyze the specific medications for IBD such as biologics [30], cumulative dosage of steroids, 5-aminosalicylates (5-ASA), and immunomodulators, such as azathioprine. Moreover, we did not investigate the role of gut microbiota which also plays a crucial role in both IBD and MAFLD.

## 5. Conclusions

Our cohort demonstrated 90% of patients with IBD and liver steatosis to be associated with metabolic dysfunction and was diagnosed with MAFLD. Additionally, we noted that MAFLD in patients with IBD was associated with shortened IBD duration, a higher Hb, an elevated GPT level, a decreased eGFR, an elevated γ-GT, and significant fibrosis. Moreover, we revealed that MAFLD was associated with elevated γ-GT and significant fibrosis. Significant fibrosis was associated with older age, as well as a noticeable trend indicating a correlation with a longer disease duration. Further studies are needed for a long-term follow-up for fibrosis progression in patients with IBD and MAFLD.

## Figures and Tables

**Table 1:** Patient baseline characteristics.

Variable	All Patients (120)	CD (45)	UC (75)	*p*-Value
Gender, Male, *n* (%)	81 (67.5%)	33 (73.3%)	48 (64.0%)	0.291
Age, year, Median (IQR)	43.5 (34–53.5)	37 (29–52)	45 (37–54)	0.089
BMI, kg/m^2^, Median (IQR)	22.6 (20.3–25.8)	22.7 (20–25.7)	22.6 (20.8–26.3)	0.584
AC, cm, Median (IQR)	81 (74–89)	82.5 (74–88)	81 (74.5–89.5)	0.807
Weight, Class *n* (%)				0.483
Underweight	12 (10.0%)	5 (11.1%)	7 (9.3%)	
Normal weight	51 (42.5%)	18 (40.0%)	33 (44.0%)	
Overweight	40 (33.3%)	18 (40.0%)	22 (29.3%)	
Obesity	17 (14.2%)	4 (8.9%)	13 (17.3%)	
IBD Duration, year, Median (IQR)	4.1 (1.4–7.5)	3.4 (2–6.1)	4.7 (1.3–9.4)	0.443
HTN, *n* (%)	17 (14.2%)	5 (11.1%)	12 (16.0%)	0.457
T2DM, *n* (%)	7 (5.8%)	3 (6.7%)	4 (5.3%)	1.000
CHB, *n* (%)	15 (12.5%)	6 (13.3%)	9 (12.0%)	0.831
CHC, *n* (%)	3 (2.5%)	1 (2.2%)	2 (2.7%)	1.000
GB Stone, *n* (%)	17 (14.2%)	12 (26.7%)	5 (6.7%)	0.002
Bowel resection history, *n* (%)	23 (19.2%)	21 (46.7%)	2 (2.7%)	<0.001
WBC count, ×10^3^/μL, Median (IQR)	6 (4.9–7.5)	6 (5–7.5)	6 (4.8–7.5)	0.519
Neutrophil-to-Lymphocyte ratio, Mean ± SD	2.87 ± 1.96	3.42 ± 2.3	2.53 ± 1.65	0.027
Hb, g/dL, Median (IQR)	13.8 (12.5–14.6)	13.4 (11.7–14.4)	14 (12.7–14.7)	0.120
Platelet count, ×10^3^/μL, Median (IQR)	261 (222–319)	257 (220–330)	268 (223–311)	0.597
ESR, mm/h, Median (IQR)	11 (5–22)	11 (5–28)	12 (5–17)	0.363
Albumin, g/dL, Median (IQR)	4.4 (4–4.6)	4.2 (3.9–4.6)	4.4 (4.1–4.6)	0.023
AST, U/L, Median (IQR)	23 (19–28)	25 (19–29)	23 (19–28)	0.552
ALT, U/L, Median (IQR)	19 (13–28)	17 (12–27)	19 (13–29)	0.295
CRP, mg/dL, Median (IQR)	0.13 (0.05–0.51)	0.24 (0.05–1.01)	0.12 (0.05–0.28)	0.045
Creatinine, mg/dL, Mean ± SD	0.81 ± 0.2	0.83 ± 0.19	0.8 ± 0.2	0.414
Cholesterol, mg/dL, Mean ± SD	166 ± 38	151 ± 37	174 ± 37	0.005
TG, mg/dL, Median (IQR)	89 (66–132)	86 (62–129)	90 (69–135)	0.429
HDL-C, mg/dL, Median (IQR)	47 (41–60)	44 (38–63)	48 (42–58)	0.571
LDL-C, mg/dL, Mean ± SD	104 ± 31	86 ± 29	113 ± 28	<0.001
ALK-P, U/L, Median (IQR)	59 (50–72)	72 (59–79)	56 (49–65)	0.003
γ-GT, U/L, Median (IQR)	16 (12–25)	16 (12–27)	17 (12–24)	0.886
Fasting glucose, mg/dL, Median (IQR)	94 (89–100)	94 (89–103)	93 (89–97)	0.887
HbA1c, %, Median (IQR)	5.5 (5.2–5.8)	5.4 (5.2–5.9)	5.5 (5.2–5.8)	0.921
Fasting insulin, μIU/mL, Median (IQR)	5.46 (3.64–9.22)	4.86 (2.92–9.22)	5.51 (3.67–9.92)	0.533
HOMA-IR, Median (IQR)	1.29 (0.86–2.23)	1.09 (0.64–2.14)	1.3 (0.86–2.5)	0.458
FIB-4, Median (IQR)	0.82 (0.55–1.31)	0.83 (0.53–1.33)	0.81 (0.57–1.29)	0.891
LSM, kPa, Median (IQR)	5.1 (4.2–5.6)	5.2 (4.1–5.6)	5.1 (4.2–5.6)	0.591
CAP, dB/m, Median (IQR)	217 (190–261)	216 (171–258)	217 (196–261)	0.432
Significant fibrosis, LSM of ≥7.2 kPa, *n* (%)	8 (6.7%)	4 (8.9%)	4 (5.3%)	0.471

Abbreviations: CD: Crohn’s disease, UC: ulcerative colitis, BMI: body mass index, AC: abdominal circumference, IBD: inflammatory bowel disease, HTN: hypertension, T2DM: type 2 diabetes mellitus, CHB: chronic hepatitis B, CHC: chronic hepatitis C, GB stone: gall bladder stone, WBC: white blood cell, Hb: hemoglobin, ESR: erythrocyte sedimentation rate, AST: aspartate aminotransferase, ALT: alanine aminotransferase, CRP: C reactive protein, TG: triglyceride, HDL-C: high density lipoprotein cholesterol, LDL-C: low density lipoprotein cholesterol, ALK-P: alkaline phosphatase, γ-GT: γ-glutamyl transpeptidase, HbA1c: glycated hemoglobin, HOMA-IR: homeostasis model assessment-insulin resistance index, FIB-4: fibrosis-4 score, LSM: liver stiffness measurement, CAP: Controlled Attenuated Parameter.

**Table 2:** Comparison of patient characteristics with and without MAFLD.

Variable	All Patients (120)	With MAFLD (35)	Without MAFLD (85)	*p*-Value
Gender, Male, *n* (%)	81 (67.5%)	28 (80.0%)	53 (62.4%)	0.061
Age, year, Median (IQR)	43.5 (34–53.5)	42 (35–52)	44 (34–55)	0.931
BMI, kg/m^2^, Median (IQR)	22.6 (20.3–25.8)	26.3 (25.1–28.6)	21.5 (19.7–23.5)	<0.001
AC, cm, Median (IQR)	81 (74–89)	93 (87–99)	76 (71–83)	<0.001
Weight Class, *n* (%)				<0.001
Under-weight	12 (10.0%)	0 (0.0%)	12 (14.1%)	0.018
Normal weight	51 (42.5%)	1 (2.9%)	50 (58.8%)	<0.001
Overweight	40 (33.3%)	22 (62.9%)	18 (21.2%)	<0.001
Obesity	17 (14.2%)	12 (34.3%)	5 (5.9%)	<0.001
IBD type, *n* (%)				0.717
UC	75 (62.5%)	21 (60.0%)	54 (63.5%)	
CD	45 (37.5%)	14 (40.0%)	31 (36.5%)	
IBD Duration, year, Median (IQR)	4.1 (1.4–7.5)	2.8 (0.6–5.1)	5.3 (2–9.4)	0.017
Anti-tumor necrosis factor α, *n* (%)	31 (25.8%)	10 (28.6%)	21 (24.7%)	0.660
Vedolizumab, *n* (%)	12 (10.0%)	2 (5.7%)	10 (11.8%)	0.505
HTN, *n* (%)	17 (14.2%)	9 (25.7%)	8 (9.4%)	0.040
T2DM, *n* (%)	7 (5.8%)	6 (17.1%)	1 (1.2%)	0.002
CHB, *n* (%)	15 (12.5%)	4 (11.4%)	11 (12.9%)	1.000
CHC, *n* (%)	3 (2.5%)	0 (0.0%)	3 (3.5%)	0.555
GB Stone, *n* (%)	17 (14.2%)	7 (20.0%)	10 (11.8%)	0.258
Bowel resection history, *n* (%)	23 (19.2%)	10 (28.6%)	13 (15.3%)	0.093
WBC count, ×10^3^/μL, Median (IQR)	6 (4.9–7.5)	6.9 (5.2–7.7)	5.7 (4.8–7.2)	0.090
Neutrophil-to-Lymphocyte ratio, Median (IQR)	2.2 (1.65–3.38)	2.31 (1.51–4.11)	2.2 (1.66–3.28)	0.670
Hb, g/dL, Median (IQR)	13.8 (12.5–14.6)	14.2 (13.4–15)	13.4 (12.2–14.5)	0.014
Platelet count, ×10^3^/μL, Median (IQR)	261 (222–319)	258 (231–307)	261 (221–323)	0.571
ESR, mm/h, Median (IQR)	11 (5–22)	11 (6–22)	12 (5–22)	0.943
Albumin, g/dL, Median (IQR)	4.4 (4–4.6)	4.4 (4.2–4.6)	4.3 (4–4.5)	0.061
AST, U/L, Median (IQR)	23 (19–28)	26 (19–31)	23 (19–28)	0.179
ALT, U/L, Median (IQR)	19 (13–28)	24 (15–34)	17 (12–25)	0.003
CRP, mg/dL, Median (IQR)	0.13 (0.05–0.51)	0.17 (0.09–0.41)	0.11 (0.04–0.63)	0.161
Creatinine, mg/dL, Mean ± SD	0.81 ± 0.2	0.91 ± 0.21	0.78 ± 0.18	0.001
eGFR, mL/min/1.73 m^2^, Mean ± SD	100.32 ± 21.14	91.37 ± 20	103.92 ± 20.61	0.004
Cholesterol, mg/dL, Mean ± SD	166 ± 38	174 ± 44	162 ± 35	0.158
TG, mg/dL, Median (IQR)	89 (66–132)	132 (86–185)	74 (57–111)	<0.001
HDL-C, mg/dL, Median (IQR)	47 (41–60)	44 (35–52)	49 (42–63)	0.026
LDL-C, mg/dL, Mean ± SD	104 ± 31	117 ± 37	98 ± 26	0.016
ALK-P, U/L, Median (IQR)	59 (50–72)	59 (55–73)	59 (49–71)	0.436
γ-GT, U/L, Median (IQR)	16 (12–25)	24 (17–49)	13 (10–21)	<0.001
Fasting glucose, mg/dL, Median (IQR)	94 (89–100)	96 (89–106)	93 (89–97)	0.154
HbA1c, %, Median (IQR)	5.5 (5.2–5.8)	5.6 (5.3–5.9)	5.4 (5.2–5.8)	0.250
Fasting insulin, μIU/mL, Median (IQR)	5.46 (3.64–9.22)	8.47 (5.56–14.17)	3.94 (3.38–6.86)	0.001
HOMA-IR, Median (IQR)	1.29 (0.86–2.23)	1.91 (1.3–3.09)	0.9 (0.77–1.76)	0.003
FIB-4, Median (IQR)	0.82 (0.55–1.31)	0.8 (0.52–1.31)	0.83 (0.57–1.25)	0.874
LSM, kPa, Median (IQR)	5.1 (4.2–5.6)	5.3 (4.7–6.1)	4.9 (4.1–5.6)	0.024
CAP, dB/m, Median (IQR)	217 (190–261)	286 (263–323)	201 (177–220)	<0.001
Significant fibrosis	8 (6.7%)	6 (17.1%)	2 (2.4%)	0.008

Abbreviations: MAFLD: metabolic dysfunction-associated fatty liver disease, BMI: body mass index, AC: abdominal circumference, IBD: inflammatory bowel disease, CD: Crohn’s disease, UC: ulcerative colitis, HTN: hypertension, T2DM: type 2 diabetes mellitus, CHB: chronic hepatitis B, CHC: chronic hepatitis C, GB stone: gall bladder stone, WBC: white blood cell, Hb: hemoglobin, ESR: erythrocyte sedimentation rate, AST: aspartate aminotransferase, ALT: alanine aminotransferase, CRP: C reactive protein, eGFR: estimated glomerular filtration rate, TG: triglyceride, HDL-C: high density lipoprotein cholesterol, LDL-C: low density lipoprotein cholesterol, ALK-P: alkaline phosphatase, γ-GT: γ-glutamyl transpeptidase, HbA1c: glycated hemoglobin, HOMA-IR: homeostasis model assessment-insulin resistance index, FIB-4: fibrosis-4 score, LSM: liver stiffness measurement, CAP: Controlled Attenuated Parameter.

**Table 3:** Multivariable analysis for factors associated with MAFLD.

Variables	Crude OR	*p*-Value	Adjusted OR	*p*-Value
Gender, Male	2.42 (0.95, 6.17)	0.065	-	-
IBD Duration	0.92 (0.85, 1.01)	0.078	-	-
Bowel resection history	2.22 (0.86, 5.68)	0.098	-	-
WBC count, ×10^3^/μL	1.16 (0.98, 1.38)	0.081	-	-
Hb, g/dL	1.33 (1.05, 1.69)	0.020	1.91 (1.17, 3.13)	0.010
Albumin, g/dL	2.72 (0.99, 7.46)	0.052	-	-
ALT, U/L	1.02 (1, 1.05)	0.060	-	-
eGFR, mL/min/1.73 m^2^	0.97 (0.95, 0.99)	0.005	-	-
LDL-C, mg/dL	1.02 (1, 1.04)	0.021	-	-
γ-GT, U/L	1.09 (1.04, 1.15)	0.001	1.11 (1.02, 1.21)	0.013
Significant fibrosis, LSM of ≥7.2 kPa, *n* (%)	8.59 (1.64, 44.94)	0.011	31.25 (1.2, 815.55)	0.039

Abbreviations: MAFLD: metabolic dysfunction-associated fatty liver disease, IBD: inflammatory bowel disease, WBC: white blood cell, Hb: hemoglobin, ALT: alanine aminotransferase, eGFR: estimated glomerular filtration rate, LDL-C: low density lipoprotein cholesterol, γ-GT: γ-glutamyl transpeptidase, LSM: liver stiffness measurement.

**Table 4:** MAFLD patient with and without significant fibrosis.

Variable	MAFLD (35)	Significant Fibrosis (6)	Nonsignificant Fibrosis (29)	*p*-Value
Gender, Male, *n* (%)	28 (80.0%)	5 (83.3%)	23 (79.3%)	1.000
Age, year, Median (IQR)	42 (35–52)	58.5 (52–64)	40 (35–48)	0.004
BMI, kg/m^2^, Median (IQR)	26.3 (25.1–28.6)	26.4 (25.2–27.8)	26.3 (25.1–28.6)	0.983
AC, cm, Median (IQR)	93 (87–99)	96.5 (93–99)	90 (86–99)	0.213
IBD disease type, *n* (%)				0.664
UC	21 (60.0%)	3 (50.0%)	18 (62.1%)	
CD	14 (40.0%)	3 (50.0%)	11 (37.9%)	
IBD Duration, year, Median (IQR)	2.8 (0.6–5.1)	4.9 (3.4–5.7)	1.6 (0.6–4.8)	0.051
Anti-tumor necrosis factor α, *n* (%)	10 (28.6%)	3 (50.0%)	7 (24.1%)	0.322
Vedolizumab, *n* (%)	2 (5.7%)	1 (16.7%)	1 (3.4%)	0.318
HTN, *n* (%)	9 (25.7%)	3 (50.0%)	6 (20.7%)	0.162
T2DM, *n* (%)	6 (17.1%)	3 (50.0%)	3 (10.3%)	0.049
Bowel resection history, *n* (%)	10 (28.6%)	3 (50.0%)	7 (24.1%)	0.322
Platelet count, ×10^3^/μL, Median (IQR)	258 (231–307)	195 (91–279)	261 (237–311)	0.049
AST, U/L, Median (IQR)	26 (19–31)	33 (21–36)	25 (19–28)	0.128
ALT, U/L, Median (IQR)	24 (15–34)	36 (26–50)	23 (15–32)	0.057
HOMA-IR, Median (IQR)	1.91 (1.3–3.09)	3.88 (3.88–3.88)	1.9 (1.3–3.05)	0.400
CAP, dB/m, Median (IQR)	286 (263–323)	289.5 (270–351)	286 (261–311)	0.460

Abbreviations: MAFLD: metabolic dysfunction-associated fatty liver disease, BMI: body mass index, AC: abdominal circumference, IBD: inflammatory bowel disease, CD: Crohn’s disease, UC: ulcerative colitis, HTN: hypertension, T2DM: type 2 diabetes mellitus, AST: aspartate aminotransferase, ALT: alanine aminotransferase, HOMA-IR: homeostasis model assessment-insulin resistance index, CAP: controlled attenuated parameter.

**Table 5:** Prevalence of NAFLD/MAFLD in the Asia-Pacific region.

	Disease Type	Fatty Liver	Number	Year	Prevalence	Diagnostic Method	Fibrosis	Diagnostic Method
China [20]	IBD	NAFLD	206	2017	10.7%	Ultrasound	-	-
Japan [19]	CD	NAFLD	303	2017	21.8%	Ultrasound	-	-
Republic of Korea [21]	IBD	NAFLD	3356	2023	16.7%	Hepatic steatosis index ≥ 30	5.3%	Fib-4 ≥ 1.45
Taiwan	IBD	MAFLD	120	2023	29.2%	TE	17.1%	LSM

Abbreviations: MAFLD: metabolic dysfunction-associated fatty liver disease, NAFLD, non-alcoholic fatty liver disease, IBD: inflammatory bowel disease, CD: Crohn’s disease, FIB-4: fibrosis-4 score, TE: transient elastography, LSM: liver stiffness measurement.

## Data Availability

The data are available on reasonable request to the corresponding author.
